# International Clinics: A Quarterly of Clinical Lectures by Professors and Lecturers in the Leading Medical Colleges of the United States, Great Britain and Canada

**Published:** 1891-09

**Authors:** 


*International Clinics: a Quarterly of Clinical Lectures by Professors and Lecturers in the leading Medical Colleges of the United States, Great Britain and Canada.* Edited by JOHN M. KEATING, M.D., J. P. CROZER GRIFFITH, M.D., J. MITCHELL BRUCE, M.D., F.R.C.P., and DAVID W. FINLAY, M.D., F.R.C.P. Volume First. Pp. viii.—357. Edinburgh: YOUNG J. PENTLAND. 1891.

As the title indicates, the publishers propose to issue quarterly a volume containing clinical lectures by the most eminent teachers in the various schools of the United States, Great Britain, and Canada. With the object of making the periodical a complete post-graduate course of medical instruction, the editors will publish only such lectures as are most instructive and most practical. Although the amount of periodical medical literature is already almost overwhelming, we think that this new publication will be a useful one, and that its special value will lie in its enabling the practitioner remote from libraries and the large centres of medical education to keep abreast with the times by learning what is the most approved practice of the leading teachers in the profession, and to compare the views and the treatment in favour at the different schools. More than this, it may be anticipated that such a periodical will serve as a further bond of union between the medical men of Great Britain and their brethren in the New World, and result in mutual improvement, by making each more perfectly acquainted with the peculiar merits of the other's practice. To take an instance, the valuable work that has been and is being done by the American Schools of Neurology and Gynæcology is not nearly so widely known in England as it deserves to be.

This first volume contains a most interesting and instructive series of Clinical Studies: where all are good it would be invidious to select any for special mention. Numerous illustrations from photographs are interspersed in the text, and serve to enable the reader—so far as a picture can do—to see for himself the case under discussion. The largest space is of course occupied by the lectures on medicine and surgery, but all the specialties find their due place.

The book is of convenient size, is printed in clear type on good paper, with well-executed illustrations, and forms a handsome volume. We must commend the enterprise of the publishers in initiating a periodical which will prove a valuable means of education to the profession; and if the succeeding volumes maintain the high level of this one—and there is every reason to believe that they will do so—we are sure that their undertaking will be crowned with success.

